# The Uremic Toxin p-Cresyl Sulfate Is a New Predictor of Major Adverse Cardiovascular Events in Patients with ST-Elevation Myocardial Infarction

**DOI:** 10.3390/toxins18010004

**Published:** 2025-12-20

**Authors:** Laure-Anne Raillon, Thomas Bochaton, Griet Glorieux, Fitsum Guebre-Egziabher, Christophe Olivier Soulage

**Affiliations:** 1 CarMeN Lab, INSERM U1060, INRAe U1397, Université Claude Bernard Lyon-1, 69500 Bron, France; laure-anne.raillon@chu-lyon.fr (L.-A.R.); thomas.bochaton@chu-lyon.fr (T.B.); fitsum.guebre-egziabher@chu-lyon.fr (F.G.-E.); 2 Hospices Civils de Lyon, Service de Néphrologie, Hôpital E Herriot, 69003 Lyon, France; 3 Hospices Civils de Lyon, Unité de Soins Intensifs Cardiologiques (USIC), Hôpital Louis Pradel, 69500 Bron, France; 4 Nephrology Section, Department of Internal Medicine and Pediatrics, Ghent University, 9000 Gent, Belgium; griet.glorieux@ugent.be

**Keywords:** ST-elevation myocardial infarction (STEMI), uremic toxins, p-cresyl sulfate (p-CS), indoxyl sulfate (IS), major adverse cardiovascular events (MACE), gut–heart axis, predictive biomarkers

## Abstract

ST-elevation myocardial infarction (STEMI) remains a major health concern despite advances in care. Indoxyl sulfate (IS) and p-cresyl-sulfate (p-CS) are gut-derived uremic toxins linked to higher morbidity and mortality in patients with chronic kidney disease (CKD). IS has been identified as an independent predictor of major adverse cardiovascular events (MACE) after STEMI, but data on p-CS are lacking. This study assessed the predictive value of IS and p-CS in STEMI patients with preserved renal function (cohort # NCT03070496). Plasma IS and p-CS were measured in 260 patients with STEMI who underwent primary coronary angiography. Samples collected 4 h after inclusion were analyzed using ultra-performance liquid chromatography with fluorescence detection. Optimal cut-offs were determined by the Youden index, and associations with MACE were evaluated by log-rank tests and Cox regression. Among 234 analyzed patients, 11.5% experienced MACE within one year. IS and p-CS levels were higher in the MACE group (IS: 3.14 vs. 2.19 µmol/L, *p* < 0.05; p-CS: 6.76 vs. 2.70 µmol/L, *p* < 0.01). Elevated p-CS independently predicted MACE (HR 3.79, 95% CI 1.29–11.17, *p* < 0.05), whereas IS lost significance after adjusting for kidney function. In STEMI patients, plasma p-CS is a stronger independent predictor of MACE than IS, highlighting its potential role in the gut–heart axis.

## 1. Introduction

ST-elevation myocardial infarction (STEMI) remains a major health issue, representing a high burden in terms of morbidity and mortality despite overall improvement in quality of care [1,2]. Consequently, it is essential to target high-risk patients following STEMI for developing optimal management approaches and treatment. Recently, the gut–heart axis has emerged as a critical actor in pathogenesis of cardiovascular disease (CVD) and is receiving growing attention [3,4,5,6]. Higher levels of gut-microbiota-derived metabolites have been associated with major adverse cardiovascular events (MACE) after STEMI, suggesting that these metabolites may be useful markers for prognosis evaluation in this clinical context [7]. Trimethylamine N-oxide (TMAO) is a gut-microbiota-derived metabolite that has been widely associated with cardio-vascular events after STEMI [8,9,10,11]. More recently, plasma phenylacetylglutamine (PAGln) and trimethyl-lysine (TML), one of the TMAO precursors, were demonstrated to be predictors of MACE in a large cohort of STEMI patients [7]. PAGln and TML were also associated with cardiovascular events and heart failure in patients with coronary artery disease [12,13,14]. Determining the most relevant gut-microbiota-derived metabolites in terms of toxicity and their association with clinical outcomes would be of great interest.

Most of these microbial metabolites accumulate during chronic kidney disease (CKD) and are responsible for many adverse biological effects. These compounds are therefore referred to as uremic toxins [15,16,17]. Indoxyl sulfate (IS) and p-cresyl sulfate (p-CS) are major gut-microbiota-derived uremic toxins produced through the microbial metabolism of dietary aromatic amino acids such as tyrosine and tryptophan. The production of these gut-microbiota-derived uremic toxins appears to be exclusively dependent on the gut microbiota since CKD-germ-free mice [18] as well as hemodialysis patients with colectomy [19] exhibit quasi-undetectable levels of IS and p-CS.

IS was associated with cardiovascular and overall mortality in CKD patients from stage 2 to 5 with a good predictive power after adjustment for the main cardiovascular risk factors. However, only plasma p-CS remained significantly and independently associated with cardiovascular events and all-cause mortality in CKD patients according to a meta-analysis conducted by Lin et al. [20]. Among PBUTs, free p-CS shows the highest association with CV outcome in non-dialysed patients with CKD [21]. Recently, two studies identified IS as an independent predictor of MACE after non-ST elevation myocardial infarction and STEMI in non-CKD patients who underwent primary coronary intervention [7,22]. Nevertheless, median baseline kidney function in these studies was significantly altered (median baseline estimated glomerular filtration rate [e-GFR] 63 and 79 mL/min/1.73 m^2^, respectively). Zwaenepoel et al. demonstrated that elevated free concentrations of IS, p-cresyl-glucuronide (p-CG), and p-CS are independently associated with an increased risk of heart failure in non-dialysed CKD patients [23]. p-CS can exert direct vascular toxicity by entering endothelial and vascular smooth muscle cells upregulating NADPH oxidase and increasing reactive oxygen species generation, which promotes endothelial dysfunction, vascular calcification, and arterial stiffness implicated in adverse cardiovascular outcomes. In endothelial cells, p-cresyl sulfate can also bind RAGE and activate NF κB signaling, downregulate flow protective transcription factors such as KLF2, and promote a pro inflammatory, pro oxidative phenotype that favors atherosclerosis, plaque instability, and thrombosis [24,25,26]. To the best of our knowledge, there is, however, no data regarding the predictive value of p-CS after STEMI. We therefore aimed to assess the predictive value of p-CS compared to that of IS in a population of STEMI patients with preserved renal function.

## 2. Results

### 2.1. Baseline Characteristics of the Population

**Figure 1:**
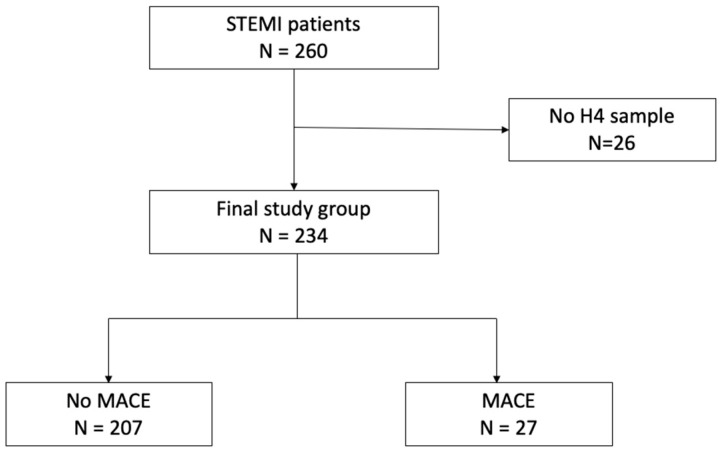
Flowchart of the study population. Abbreviations: MACE, major adverse cardiovascular event, STEMI, ST-elevation myocardial infarction.

A total of 234 patients with STEMI were included in the analysis, after excluding 26 patients for whom IS and p-CS levels could not be assessed due to the unavailability of the sample collected 4 h post admission (thereafter referred to as H4 sample) (Figure 1). Baseline characteristics of the whole population and of the subgroups with or without MACE are presented in Table 1. The median [IQR] age was 57.9 [51–66.4] years, and 79.9% of the patients were males. Patients were significantly older (74.4 vs. 57.1 years, *p* < 0.001) in the MACE group, and the proportion of patients with diabetes was higher compared to the no-MACE group (32.1 vs. 13.2%, respectively, *p* = 0.01). Cardiac biomarker levels (B-type natriuretic peptide, BNP and High-Sensitivity Cardiac Troponin I, hs-cTnI) were significantly higher in the MACE group. Median eGFR at admission in the total population was high (97 mL/min.1.73 m^2^) but lower in the MACE group compared to the no-MACE group (72 vs. 99 mL/min.1.73 m^2^, *p* < 0.01). Regarding renal function, 13 patients exhibited eGFR < 60 (i.e., with CKD), 58 patients had eGFR between 60 and 89 (i.e., with mild to moderate CKD), and 163 had an eGFR > 90 mL/min.1.73 m^2^.

**Figure 2:**
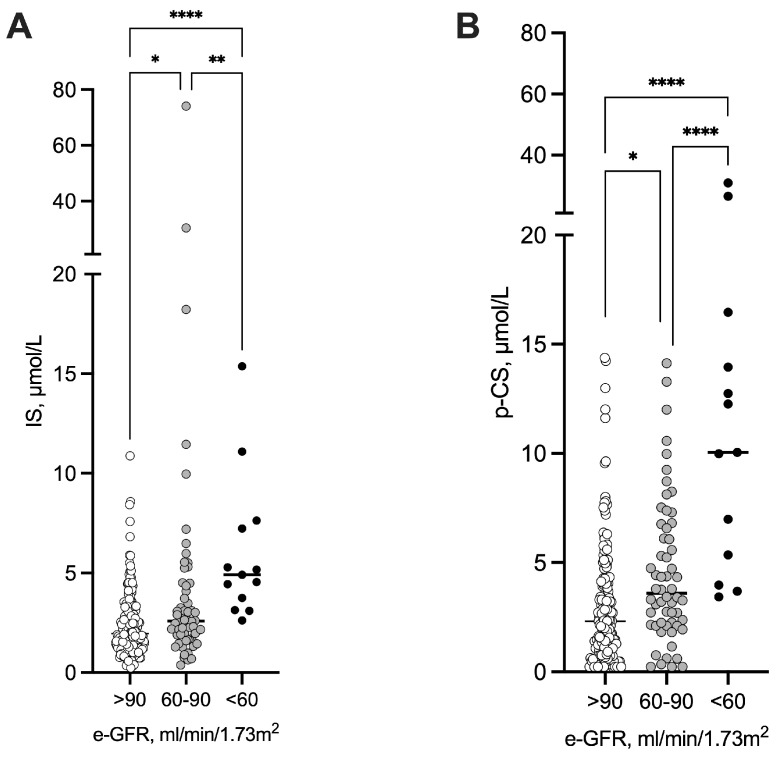
Indoxyl sulfate and p-cresyl sulfate levels according to baseline kidney function. Differences were considered significant at the *p* < 0.05 level. * *p* < 0.05, ** *p* < 0.01, **** *p* < 0.001. Abbreviations: e-GFR, estimated glomerular filtration rate; IS, indoxyl sulfate; p-CS, p-cresyl sulfate.

There was no significant difference in suboptimal treatment prescription between the two groups. The levels of IS and p-CS were inversely correlated with baseline kidney function; surprisingly, both levels were significantly higher as soon as eGFR was <90 mL/min.1.73 m^2^ (Figure 2). The plasma concentrations of other uremic toxins are shown in Supplementary Table S1.

### 2.2. STEMI Patients with MACE Exhibited Higher Levels of IS and p-CS

**Figure 3:**
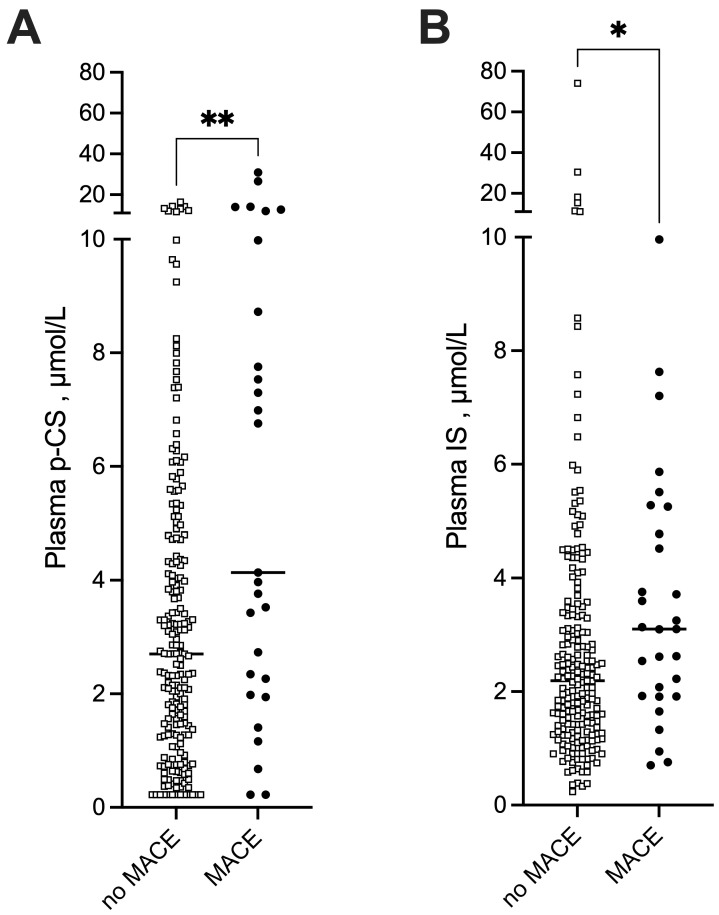
(**A**) p-cresyl sulfate (p-CS) and (**B**) indoxyl sulfate (IS) levels in patients with and without major adverse cardiovascular events (MACE). Differences were considered significant at the p < 0.05 level. *, p < 0.05, ** p < 0.01. Abbreviations: IS, indoxyl-sulfate, MACE, major adverse cardiovascular event, p-CS, p-cresyl-sulfate.

**Figure 4:**
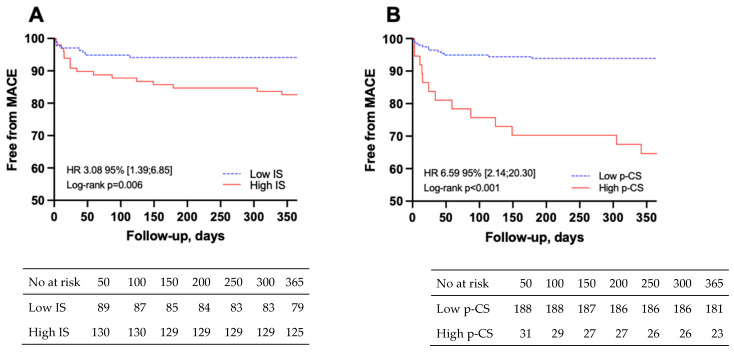
High indoxyl sulfate (IS) and p-cresyl sulfate (p-CS) levels predict major adverse cardiovascular events (MACE). Cut-offs were determined using the Youden index of ROC curves. (**A**) Kaplan–Meier curve for indoxyl sulfate (cut-off = 2.52 µmol/L); (**B**) Kaplan–Meier curve for p-cresyl sulfate (cut off = 6.67 µmol/L).

During the one-year follow-up, 27 STEMI patients (11.5%) experienced a MACE: 5 deaths (18.5%), 3 strokes (11.1%), 13 new or worsening heart failure (48.0%), 6 non-fatal reinfarction (22.2%). IS and p-CS concentrations were higher in the MACE group than in the no-MACE group, with a median concentration of IS of 3.14 [1.92–5.25] vs. 2.19 [1.37–3.35] µmol/L (*p* < 0.05) and a median concentration of p-CS of 6.76 [2.35–10.60] vs. 2.70 [1.28–4.72] µmol/L (*p* < 0.01), respectively (Figure 3). According to the Youden index of ROC curves, the optimal cutoff values to predict MACE in patients with STEMI were 2.52 µmol/L for plasma IS (sensitivity 68% and specificity 61%) and 6.67 µmol/L for plasma p-CS (sensitivity 52% and specificity 89%). Kaplan–Meier survival analysis demonstrated that STEMI patients with higher plasma IS and higher plasma p-CS exhibited significantly greater risks of MACE than those with lower concentrations (log-rank, *p* = 0.006 and *p* < 0.001, respectively, Figure 4). The details of the specific MACEs (e.g., death, non-fatal reinfarction, exacerbation of heart failure, stroke) observed in each group are presented in Supplementary Table S2. Notably, no difference was observed between patients with low and high p-CS levels (Fisher’s exact test, *p* = 0.15).

### 2.3. Plasma p-CS Is an Independent Predictor of MACE After STEMI

In univariate analysis, age, diabetes, peak hs-cTnI level, BNP, eGFR levels at admission, and high concentrations of IS and p-CS were significant predictors of MACE. Sex and a history of hypertension showed a trend without reaching statistical significance (Table 2). According to the Cox regression analysis, high plasma levels of p-CS remained significantly associated with MACE after adjustment for all risk factors identified in the univariate analysis with an HR of 3.79 95%CI [1.29; 11.17] (*p* < 0.05). High plasma levels of IS remained significantly associated with MACE after adjusting for STEMI patient medical history and laboratory cardiac data such as peak hs-cTnI and BNP at admission (HR 2.49 [1.05;5.91], *p* < 0.05 and HR 2.79 [1.13; 6.85], *p* < 0.05, respectively). However, high plasma levels of IS did not remain significantly associated with MACE when adjusted for admission eGFR, with an HR of 1.28 [0.78; 3.46] (*p* = 0.6; Table 3).

## 3. Discussion

Over the last few years, there has been an increasing interest in the gut–heart axis [27,28]. In the present study, we investigated the association between plasma concentration of two gut-derived uremic toxins (IS and p-CS) and MACE in patients with STEMI. We found that higher plasma p-CS was an independent predictor of MACE in patients with STEMI, with a better prognostic value than IS. Indeed, p-CS remained significantly associated with MACE after adjusting for all identified risk factors. In contrast, IS remained a predictor of MACE after adjusting for cardiac data but not after adjusting for baseline kidney function. To our knowledge, this is the first study investigating the association between plasma p-CS and MACE after STEMI. The present results suggest that, although IS has more often raised scientific interest, p-CS could represent a better gut–heart axis marker for prognosis evaluation after STEMI. Interestingly, free p-CS has already been associated with cardiovascular events independently of eGFR and of Framingham risk factors in CKD patients [29] and in patients in hemodialysis [30]. The direct toxicity of p-CS, however, remains questionable in the present study. Indeed, the p-CS concentration reported herein, in a population with preserved kidney function, is about 15 times lower than that observed in patients with end-stage kidney disease (around 110 µmol/L) [15], in whom p-CS toxicity and association with cardiovascular events and mortality is already well described. At this level, p-CS is associated with vascular inflammation, calcification, and atherogenesis in patients with CKD [17]. This involves an induction of oxidative stress [24], increased expression of pro-inflammatory factors such as MCP-1 and TNF-α [31] or activation of NF-κB [32], and the overexpression of adhesion molecules such as E-selectin, ICAM-1, and VCAM-1 [31]. The p-CS levels observed herein could thus indicate an altered systemic environment that promotes the occurrence of MACE rather than a direct toxic effect. IS and p-CS are end-products of bacterial fermentation of tryptophan and tyrosine in the colon [19]. Their increased levels in the MACE group could be related to an exacerbated intestinal generation resulting from dietary intakes and/or increase absorption over the intestinal barrier and gut dysbiosis.

It was recently shown that phenolic compounds (such as p-cresol) are mostly generated by anaerobic bacteria whereas indolic compounds (such as indole) are produced by both anaerobes and aerobes [33]. The generation of p-CS could thus be an intermediate related to different dietary habits or a substantial inflammatory state linked to dysbiosis that could subsequently favor the occurrence of MACE. It should, however, be noted that these mechanisms remain hypothetical and require further experimental evidence. Information regarding diet, gut microbiome characteristics, use of antibiotics or probiotics, and gastrointestinal symptoms such as diarrhea or abdominal pain prior to STEMI was unfortunately not recorded in our cohort to support this hypothesis.

Data from [21] suggests that uremic toxins with normal kidney function might also have a clinical impact, since pCS remains significantly associated with CVD after adjustment for multiple risk factors including eGFR. Many inflammatory and anti-inflammatory biomarkers have already been assessed in the HIBISCUS STEMI cohort [34,35]. IS and p-CS showed only weak associations (rs < 0.3) with these biomarkers (see Supplementary Table S3). For example, IS was positively correlated with interleukin-6 (IL-6), IL-8, IL-10, and monocyte chemotactic protein 1 (MCP-1), while p-CS was positively correlated with IL-6, IL-10, and Interleukin 1 receptor-like 1 (ST-2). The strength of the present study lies in the cohort and prospective design of the initial study. First, the cohort is representative of a non-selected population of patients with STEMI and preserved kidney function. The study population exhibited better kidney function than in previous studies investigating the predictive value of IS in non-ST and STEMI populations [7,22] (median baseline eGFR 63 and 79, respectively, vs. 97 mL/min.1.73 m^2^ in the present cohort), limiting the confounding effect of baseline kidney function on the occurrence of MACE. Furthermore, all patients underwent a standardized treatment (PCI) and all had a complete follow up. Finally, the early sampling performed at 4 h after admission highlights that p-CS elevation could be used as an early prognostic marker, enabling to improve prompt management of the patients. However, p-CS and IS assays are not currently performed in clinical routine practice. A limitation of the present study is its single-center nature, possibility limiting the generalizability of the findings. The very small number of patients with MACE (27 cases, approximately 10% of the cohort) should also be acknowledged as a limitation of the study as it limits the statistical power. The limited number of patients with an eGFR < 60 mL/min.1.73 m^2^ (13 cases, i.e., roughly 5% of the cohort) does not allow us to perform subgroup analysis in this population. Furthermore, the single-center design may reduce generalizability. Due to the limited sample volume available, only the total fraction of p-CS could be assayed, whereas its toxicity is most likely due to the free fraction (>90% protein binding). Because only total p-CS could be measured in our study, and free p-CS is considered the only biologically active fraction, our findings may underestimate the true toxic effects of this uremic toxin, representing an important limitation of our analysis. Unfortunately, information on patient albumin levels was lacking, which could have been interesting assuming that a low level of plasma albumin could be responsible for an enhanced p-CS free fraction and toxicity. However, the study by Verbeke et al. [36] demonstrates that the association of PBUTs with cardiovascular risk is not explained by albumin levels, and that PBUTs remains a strong and independent predictor for adverse outcome regardless of albuminemia. Although routine measurement of p-CS is not yet part of clinical practice, our findings suggest that p-CS could eventually serve as an early risk-stratification tool in STEMI management. Because elevated p-CS levels independently predicted 1-year MACE in patients with preserved renal function, integrating this biomarker into future clinical pathways could help identify high-risk individuals who may benefit from intensified monitoring or adjunctive therapies. Further validation in larger cohorts and the development of rapid, clinically compatible assays will be essential steps toward translating p-CS measurement into actionable bedside decision-making.

## 4. Conclusions

Plasma total p-CS concentration is an independent predictor of MACE in STEMI patients (HR 3.79, 95% CI 1.29–11.17) with preserved kidney function. Gut-derived metabolites could represent an intermediate on the gut–heart axis reflecting a dysbiotic state favoring MACE occurrence after STEMI. Further dedicated studies should investigate the precise role of p-CS in this clinical context by assessing patients’ dietary habits (especially protein and fiber intakes), serum albumin, and composition of the host microbiome to better understand the relation between plasma p-CS levels and MACE. These findings, however, need to be further validated in larger multicentric cohorts.

## 5. Materials and Methods

### 5.1. Study Population

The present study is based on a post hoc analysis of the HIBISCUS-STEMI trial database (registered in Clinicaltrials.gov under the reference NCT03070496) and serum biobank (NeuroBioTec biobank, CRB HCL: BB-0033-00046, Bron, France). HIBISCUS-STEMI is a prospective cohort that included, from 2016 to 2019, 260 consecutive patients with STEMI who underwent revascularization during primary coronary intervention (PCI) at the Hôpital Louis Pradel (Hospices Civils de Lyon, Lyon, France), a tertiary referral university hospital. STEMI was defined by the presence of clinical symptoms (chest pain) associated with an ST elevation of more than 2 mm in two contiguous leads on a standard 12-lead electrocardiogram. Patients without STEMI diagnosis confirmation on angiography or with magnetic resonance imaging (MRI) contraindication were excluded. Patients were enrolled in the study irrespective of their renal status at admission. The study was approved by the local research ethics committee (CPP Sud-Est III, number: 2015-067B, date: 19 May 2015) and conducted according to the Declaration of Helsinki. Written informed consent was obtained from all participants before inclusion in the study.

### 5.2. Clinical Data

Demographic and clinical data were collected for each patient at admission and prospectively recorded during systematic follow-up visits up to 1 year after STEMI. MACEs were defined as a composite of death, non-fatal reinfarction, hospitalizations for new or worsening heart failure, and stroke. Suboptimal treatment was defined as the absence of at least one of the following drug classes at discharge or 1-month follow up: thienopyridines, acetyl salicylic acid, angiotensin-converting enzyme inhibitor (ACEI) or angiotensin receptor blocker (ARB), β-blocker, and/or statin. Serum from all patients was collected at five time points: admission and 4 h (H4), 24 h (H24), 48 h (H48), and 1 month (M1) after PCI. Measurement of uremic toxins was only performed on the H4 sample.

### 5.3. Laboratory Data

Brain natriuretic peptides (BNP), high-sensitivity cardiac troponin I (hs-cTnI), and serum creatinine were routinely measured at admission and during follow-up using a standard enzymatic assay in a centralized laboratory. Baseline eGFR was calculated using the Chronic Kidney Disease Epidemiology Collaboration (CKD EPI) formula [37]. Acute kidney injury was assessed according to the Acute Kidney Injury Network (AKIN) definition [38]. Blood samples were collected 4 h after inclusion (H4), centrifuged (2000× *g*, 15 min), and plasma were stored at −80 °C in the hospital biobank (NeuroBioTec Biological Resource Center, Bron, France). Total plasma concentrations of uremic toxins, including p-CS and IS, were assayed by reverse-phase ultra performance liquid chromatography (RP-UPLC, Agilent 1290 Infinity, Agilent, Santa Clara, CA, USA) coupled with fluorescence detection (pCS: λ_exc._ 265 nm/λ_em_ 290 nm, IS: λ_exc._ 280 nm/λ_em_ 340 nm) as previously described [21,39,40]. The limit of detection (LOD) and limit of quantification (LOQ) for p-CS were 0.32 and 1.06 µmol/L, respectively. The LOD and LOQ for IS were 0.02 and 0.07 µmol/L, respectively.

### 5.4. Statistical Analysis

Data were checked for normality using QQ plot and Shapiro–Wilk test. As all numeric data (except age) were not normally distributed, data were expressed as median and interquartile range (IQR). Categorical data were expressed as numbers and percentages. The population was divided into two subgroups: the MACE group and the no-MACE group. Comparisons of baseline characteristics between the MACE and no-MACE groups were performed using unpaired *t*-test or Mann–Whitney test for continuous variables and Pearson χ^2^ test or Fisher’s exact test for categorical variables. The optimal cutoff values of plasma concentrations of IS and p-CS to predict MACE were determined according to the Youden index from receiver operating characteristic (ROC) curves. Time-to-event analysis according to high or low IS and p-CS was assessed using the Kaplan–Meier curve and differences were tested with the log-rank test. The association of IS and p-CS levels with MACE was further explored using different Cox regression models including all risk factors identified in the univariate analysis with a *p*-value ≤ 0.1 or other well-known risk factors. Several models with a limited number of predictors (fewer than three) were run to promote parsimony and reduce the risk of overfitting. Model 1 was adjusted for demographic characteristics—age and sex—because mean age differed between groups (*p* < 0.001) and male sex is a well-known cardiovascular risk factor. Model 2 was adjusted for patients’ medical history, including diabetes mellitus (*p* = 0.01), hypertension (borderline significant, *p* = 0.09), and dyslipidemia (non-significant, *p* = 0.73), as all are recognized cardiovascular risk factors. Model 3 was adjusted for laboratory biomarkers of cardiac injury or heart failure, specifically peak hs-I troponin (*p* = 0.09) and BNP at admission (*p* < 0.001). Model 4 was adjusted for estimated glomerular filtration rate at admission (eGFR, *p* = 0.01), given that concentrations of uremic toxins are strongly dependent on renal function. Statistical significance was defined as a *p*-value < 0.05. All statistical analyses were performed using Graphpad Prism 10.6.1 (2025) (Graphpad software, La Jolla, CA, USA) and open-source R 4.5.1 (https://www.r-project.org/) software using the Jamovi 2.3.28.0 front end (https://www.jamovi.org/).

## Figures and Tables

**Table 1:** Demographic, clinical, and laboratory characteristics of STEMI patients according to the occurrence of major adverse cardiovascular events (MACE).

	Total Population (*n* = 234)	No MACE (*n* = 207, 87.6%)	MACE (*n* = 27, 11.5%)	*p*-Value
**Baseline characteristics**				
Age, year	57.9 [51–66.4] {2}	57.1 [50.5–63.3] {2}	74.4 [66.6–78.8] {0}	<0.001
Female, *n* (%)	47 (20.1) {0}	39 (18.7) {0}	8 (32) {0}	0.12
BMI, kg/m^2^	26.2 [23.8–29.4] {11}	26.2 [23.7–29.4] {10}	26.3 [25–29.5] {1}	0.51
Hypertension, *n* (%)	69 (29.5) {9}	58 (28.9) {8}	11 (45.8) {1}	0.09
Current smoker, *n* (%)	118 (50.4) {8}	110 (55.6) {7}	8 (28.6) {1}	0.01
Dyslipidemia, *n* (%)	68 (29) {9)	60 (29.9) {8}	8 (33.3) {1}	0.73
Diabetes mellitus, *n* (%)	35 (15) {9}	26 (13.2) {8}	9 (32.1) {1}	0.01
**Cardiologic clinical and biological setting**				
Systolic BP, mmHg	134 [116–151] {17}	134 [116–150] {14}	141 [119–159] {3}	0.27
Diastolic BP, mmHg	85 [70–96] {17}	85 [70–96] {14}	83 [75–105] {3}	0.45
HR, bpm	75 [64–87] {19}	74 [64–86] {15}	78 [65–93] {4}	0.43
Killip stage ≥ 2, *n* (%)	8 (3.8) {23}	6 (3.2) {22}	2 (8.3) {1}	0.23
Ischemic time, min	200 [142–309] {55}	195 [145–302] {49}	217 [128–345] {6}	0.70
Contrast medium volume, mL	90 [66–116] {1}	90 [66–115] {1}	95 [62–120] {0}	0.98
Admission LVEF, %	55 [45–61] {117}	55 [46–61] {104}	58.5 [40.5–52.8] {13}	0.86
Infarct size on 1-month MRI, % of LV mass	14.3 [7–24.4] {71}	14.3 [6.7–24] {55}	17.2 [8.7–29.8] {16}	0.24
BNP at admission, ng/L	33 [15–81.5] {19}	29.5 [14.8–76.3] {17}	81 [40–236] {2}	<0.001
hs-I Troponine at admission, µg/L	297 [76–1506] {5}	282 [68–1478] {4}	618 [211–1728] {1}	0.09
Peak hs-I Troponine, mg/L	43.6 [14.0–110.7] {0}	40.2 [13.1–99.2] {0}	122.3 [34.7–199.5] {0}	0.001
CRP at admission, mg/L	3.30 [1.70–7.6] {11}	3.15 [1.7–7.1] {11}	4.60 [2.5–10.1] {0}	0.07
**Renal function**				
Creatinine at admission, µmol/L	70.5 [61–83] {0}	70 [61–81] {0}	82 [63–103] {0}	0.05
Creatinine at H48, µmol/L	80.5 [71–91.8] {0}	80 [71–89] {0}	83 [71–134] {0}	0.22
Admission eGFR, mL/min/1.73 m^2^	97.2 [86.3–106] {0}	98.8 [88.8–106] {0}	71.9 [59.9–93.7] {0}	<0.01
Admission eGFR < 90 mL/min/1.73 m^2^, *n* (%)	75 (32) {0}	59 (28.2) {0}	16 (64) {0}	<0.01
Admission eGFR < 60 mL/min/1.73 m^2^, *n* (%)	14 (6) {0}	7 (3.3) {0}	7 (8) {0}	<0.01
**Suboptimal treatment**				
Suboptimal treatment at discharge, *n* (%)	58 (25.8) {9}	51 (25.2) {7}	7 (30.4) {2}	0.59
Suboptimal treatment at 1-month, *n* (%)	47 (25.4) {49}	42 (24.9) {40}	5 (31.3) {9}	0.56

Continuous values are represented as median [IQR], {number of missing data}. Categorical data are represented as number (%), {number missing data}. Suboptimal treatment was defined as the absence of at least one of the following drug classes: thienopyridines, acid acetyl acetic, angiotensin-converting enzyme inhibitor or angiotensin receptor blocker, β-blocker, statin. Abbreviations: BMI, body mass index; BP, blood pressure; HR, heart rate; PCI, Percutaneous coronary intervention; LVEF, left ventricle ejection fraction; MRI, magnetic resonance imaging; BNP, B type natriuretic peptide; CRP, C reactive protein; eGFR, estimated glomerular filtration rate.

**Table 2:** Univariate analysis of predictive factors of MACE in a cohort of STEMI patients.

	Univariate Analysis	
	HR [95% CI]	*p*-Value
Age	1.10 [1.07; 1.12]	<0.001
Sex (Female)	2.04 [0.88; 4.72]	0.10
BMI	1.03 [0.94; 1.14]	0.52
Hypertension	1.99 [0.89; 4.43]	0.09
Dyslipidemia	1.14 [0.49; 2.66]	0.76
Diabetes mellitus	3.46 [1.52; 7.92]	0.003
Ischemic time	1 [0.99; 1.00]	0.75
peak hs-I Troponine, log	1.75 [1.23; 2.49]	0.002
BNP at admission, log	1.82 [1.31; 2.53]	<0.001
LVEF at admission	0.99 [0.94; 1.04]	0.68
Infarct size on 1-month MRI, % of LV mass	1.03 [0.99; 1.07]	0.15
CRP at admission	0.99 [0.97; 1.02]	0.73
eGFR at admission	0.96 [0.94; 0.97]	<0.001
IS > 2.52 µmol/L	3.02 [1.30; 7.01]	0.01
p-CS > 6.67 µmol/L	6.63 [3.02; 14.55]	<0.001

CI, confidence interval; HR, hazard ratio; BMI, body mass index; BNP, Brain natriuretic peptide; LVEF, left ventricule ejection fraction; MRI, magnetic resonance imaging; CRP, C reactive protein; eGFR, estimated glomerular filtration rate; IS, indoxyl sulfate; p-CS, p-cresyl sulfate.

**Table 3:** Multivariable logistic regression analysis of IS and p-CS as predictors of major adverse cardiovascular events after STEMI.

	HR (95% CI)	*p*-Value
**p-CS > 6.67 µmol/L**		
Unadjusted	6.63 [3.02; 14.55]	<0.001
Model 1 ^a^	3.33 [1.40; 7.89]	0.006
Model 2 ^b^	6.19 [2.71; 14.16]	<0.001
Model 3 ^c^	5.15 [2.21; 12.01]	<0.001
Model 4 ^d^	3.15 [1.26; 7.86]	0.01
**IS > 2.52 µmol/L**		
Unadjusted	3.02 [1.30; 7.01]	0.01
Model 1 ^a^	1.84 [0.75; 4.55]	0.19
Model 2 ^b^	2.49 [1.05; 5.91]	0.04
Model 3 ^c^	2.79 [1.13; 6.85]	0.03
Model 4 ^d^	1.28 [0.78; 3.46]	0.62

CI, confidence interval; HR, hazard ratio; p-CS, p-cresyl sulfate; IS, indoxyl sulfate. ^a^ Model 1 was adjusted for demographic characteristics: age, sex. ^b^ Model 2 was adjusted for patients’ medical history: diabetes mellitus, hypertension, dyslipidemia. ^c^ Model 3 was adjusted for laboratory cardiac data: peak hs-I Troponine (log), BNP at admission (log). ^d^ Model 4 was adjusted for admission estimated glomerular filtration rate (eGFR).

## Data Availability

The datasets generated during the current study are not publicly available but could be made available from the corresponding author on reasonable request. The data are not publicly available due to regulatory and ethical restrictions.

## Supplementary Materials

The following supporting information can be downloaded at: https://www.mdpi.com/article/10.3390/toxins18010004/s1, Supplementary Table S1: Total plasma concentration of other uremic toxins in STEMI patients according to the occurrence of major cardiovascular events (MACE); Supplementary Table S2: Detail of the 27 major adverse cardiovascular events (MACE) encountered by STEMI patients during the follow-up period; Supplementary Table S3: Correlation between uremic toxin levels and inflammatory biomarkers in STEMI patients; Supplementary Figure S1: High indoxyl sulfate (IS) and p-cresyl sulfate (p-CS) levels predict MACE in the sub-population of STEMI patients with eGFR ≥ 60 mL/min.1.73 m^2^.

## Abbreviations

The following abbreviations are used in this manuscript:

BMI	Body mass index
BNP	B type natriuretic peptide
BP	Blood pressure
95%CI	95% confidence interval
CKD	Chronic kidney disease
CRP	C reactive protein
eGFR	Estimated glomerular filtration rate
HR	Heart rate
HSP-70	70 kilodalton heat shock proteins
hs-cTnI	High-Sensitivity Cardiac Troponin I
IL-6	interleukin-6
IL-8	interleukin-8
IL-10	interleukin-10
IQR	Interquartile range
IS	Indoxyl-sulfate
LVEF	Left ventricle ejection fraction
PBUTs	Protein-bound uremic toxins
MACE	Major adverse cardiovascular event
MCP-1	monocyte chemoattractant protein 1
MRI	Magnetic resonance imaging
PAGln	Phenylacetylglutamine
p-CG	p-cresyl-glucuronide
p-CS	p-cresyl-sulfate
PCI	Percutaneous coronary intervention
ROC	Receiver Operating Characteristic
ST2	Interleukin 1 receptor-like 1
STEMI	ST-elevation myocardial infarction
TMAO	Trimethylamine N-oxide
TML	Trimethyl-lysine

## References

[1] Fox K.A.A., Eagle K.A., Gore J.M., Steg P.G., Anderson F.A., GRACE and GRACE2 Investigators (2010). The Global Registry of Acute Coronary Events, 1999 to 2009—GRACE. Heart Br. Card. Soc..

[2] Tsao C.W., Aday A.W., Almarzooq Z.I., Alonso A., Beaton A.Z., Bittencourt M.S., Boehme A.K., Buxton A.E., Carson A.P., Commodore-Mensah Y. (2022). Heart Disease and Stroke Statistics—2022 Update: A Report from the American Heart Association. Circulation.

[3] Zhang Y., Wu H., Jin M., Feng G., Wang S. (2025). The Gut-Heart Axis: Unveiling the Roles of Gut Microbiota in Cardiovascular Diseases. Front. Cardiovasc. Med..

[4] Bui T.V.A., Hwangbo H., Lai Y., Hong S.B., Choi Y.-J., Park H.-J., Ban K. (2023). The Gut-Heart Axis: Updated Review for The Roles of Microbiome in Cardiovascular Health. Korean Circ. J..

[5] Snelson M., Muralitharan R.R., Liu C.-F., Markó L., Forslund S.K., Marques F.Z., Tang W.H.W. (2025). Gut-Heart Axis: The Role of Gut Microbiota and Metabolites in Heart Failure. Circ. Res..

[6] Rivera K., Gonzalez L., Bravo L., Manjarres L., Andia M.E. (2024). The Gut–Heart Axis: Molecular Perspectives and Implications for Myocardial Infarction. Int. J. Mol. Sci..

[7] Zhao S., Tian Y., Wang S., Yang F., Xu J., Qin Z., Liu X., Cao M., Zhao P., Zhang G. (2023). Prognostic Value of Gut Microbiota-Derived Metabolites in Patients with ST-Segment Elevation Myocardial Infarction. Am. J. Clin. Nutr..

[8] Wang Z., Klipfell E., Bennett B.J., Koeth R., Levison B.S., Dugar B., Feldstein A.E., Britt E.B., Fu X., Chung Y.-M. (2011). Gut Flora Metabolism of Phosphatidylcholine Promotes Cardiovascular Disease. Nature.

[9] Li X.S., Obeid S., Klingenberg R., Gencer B., Mach F., Räber L., Windecker S., Rodondi N., Nanchen D., Muller O. (2017). Gut Microbiota-Dependent Trimethylamine N-Oxide in Acute Coronary Syndromes: A Prognostic Marker for Incident Cardiovascular Events beyond Traditional Risk Factors. Eur. Heart J..

[10] Vakadaris G., Korovesis T., Balomenakis C., Papazoglou A.S., Papadakos S.P., Karniadakis I., Moysidis D.V., Arvanitakis K., Germanidis G., Brilakis E.S. (2025). Prognostic Value of Serum TMAO Measurement in Patients with STEMI: A Systematic Literature Review. Curr. Vasc. Pharmacol..

[11] Tang W.H.W., Wang Z., Levison B.S., Koeth R.A., Britt E.B., Fu X., Wu Y., Hazen S.L. (2013). Intestinal Microbial Metabolism of Phosphatidylcholine and Cardiovascular Risk. N. Engl. J. Med..

[12] Liu Y., Liu S., Zhao Z., Song X., Qu H., Liu H. (2021). Phenylacetylglutamine Is Associated with the Degree of Coronary Atherosclerotic Severity Assessed by Coronary Computed Tomographic Angiography in Patients with Suspected Coronary Artery Disease. Atherosclerosis.

[13] Romano K.A., Nemet I., Prasad Saha P., Haghikia A., Li X.S., Mohan M.L., Lovano B., Castel L., Witkowski M., Buffa J.A. (2023). Gut Microbiota-Generated Phenylacetylglutamine and Heart Failure. Circ. Heart Fail..

[14] Li X.S., Obeid S., Wang Z., Hazen B.J., Li L., Wu Y., Hurd A.G., Gu X., Pratt A., Levison B.S. (2019). Trimethyllysine, a Trimethylamine N-Oxide Precursor, Provides near- and Long-Term Prognostic Value in Patients Presenting with Acute Coronary Syndromes. Eur. Heart J..

[15] Duranton F., Cohen G., De Smet R., Rodriguez M., Jankowski J., Vanholder R., Argiles A. (2012). on behalf of the European Uremic Toxin Work Group. Normal and Pathologic Concentrations of Uremic Toxins. J. Am. Soc. Nephrol..

[16] Vanholder R., Baurmeister U., Brunet P., Cohen G., Glorieux G., Jankowski J. (2008). on behalf of the European Uremic Toxin Work Group. A Bench to Bedside View of Uremic Toxins. J. Am. Soc. Nephrol..

[17] Lim Y.J., Sidor N.A., Tonial N.C., Che A., Urquhart B.L. (2021). Uremic Toxins in the Progression of Chronic Kidney Disease and Cardiovascular Disease: Mechanisms and Therapeutic Targets. Toxins.

[18] Mishima E., Fukuda S., Mukawa C., Yuri A., Kanemitsu Y., Matsumoto Y., Akiyama Y., Fukuda N.N., Tsukamoto H., Asaji K. (2017). Evaluation of the Impact of Gut Microbiota on Uremic Solute Accumulation by a CE-TOFMS-Based Metabolomics Approach. Kidney Int..

[19] Aronov P.A., Luo F.J.-G., Plummer N.S., Quan Z., Holmes S., Hostetter T.H., Meyer T.W. (2011). Colonic Contribution to Uremic Solutes. J. Am. Soc. Nephrol..

[20] Lin C.-J., Wu V., Wu P.-C., Wu C.-J. (2015). Meta-Analysis of the Associations of p-Cresyl Sulfate (PCS) and Indoxyl Sulfate (IS) with Cardiovascular Events and All-Cause Mortality in Patients with Chronic Renal Failure. PLoS ONE.

[21] Glorieux G., Vanholder R., Van Biesen W., Pletinck A., Schepers E., Neirynck N., Speeckaert M., De Bacquer D., Verbeke F. (2021). Free P-Cresyl Sulfate Shows the Highest Association with Cardiovascular Outcome in Chronic Kidney Disease. Nephrol. Dial. Transplant. Off. Publ. Eur. Dial. Transpl. Assoc.-Eur. Ren. Assoc..

[22] Watanabe I., Tatebe J., Fujii T., Noike R., Saito D., Koike H., Yabe T., Okubo R., Nakanishi R., Amano H. (2019). Prognostic Utility of Indoxyl Sulfate for Patients with Acute Coronary Syndrome. J. Atheroscler. Thromb..

[23] Zwaenepoel B., De Backer T., Glorieux G., Verbeke F. (2024). Predictive Value of Protein-Bound Uremic Toxins for Heart Failure in Patients with Chronic Kidney Disease. ESC Heart Fail..

[24] Watanabe H., Miyamoto Y., Enoki Y., Ishima Y., Kadowaki D., Kotani S., Nakajima M., Tanaka M., Matsushita K., Mori Y. (2015). P-Cresyl Sulfate, a Uremic Toxin, Causes Vascular Endothelial and Smooth Muscle Cell Damages by Inducing Oxidative Stress. Pharmacol. Res. Perspect..

[25] Wu P.-H., Lin Y.-T., Chiu Y.-W., Baldanzi G., Huang J.-C., Liang S.-S., Lee S.-C., Chen S.-C., Hsu Y.-L., Kuo M.-C. (2021). The Relationship of Indoxyl Sulfate and P-Cresyl Sulfate with Target Cardiovascular Proteins in Hemodialysis Patients. Sci. Rep..

[26] Harlacher E., Wollenhaupt J., Baaten C.C.F.M.J., Noels H. (2022). Impact of Uremic Toxins on Endothelial Dysfunction in Chronic Kidney Disease: A Systematic Review. Int. J. Mol. Sci..

[27] Lupu V.V., Adam Raileanu A., Mihai C.M., Morariu I.D., Lupu A., Starcea I.M., Frasinariu O.E., Mocanu A., Dragan F., Fotea S. (2023). The Implication of the Gut Microbiome in Heart Failure. Cells.

[28] Trøseid M., Andersen G.Ø., Broch K., Hov J.R. (2020). The Gut Microbiome in Coronary Artery Disease and Heart Failure: Current Knowledge and Future Directions. EBioMedicine.

[29] Meijers B.K.I., Claes K., Bammens B., de Loor H., Viaene L., Verbeke K., Kuypers D., Vanrenterghem Y., Evenepoel P. (2010). P-Cresol and Cardiovascular Risk in Mild-to-Moderate Kidney Disease. Clin. J. Am. Soc. Nephrol..

[30] Meijers B.K.I., Bammens B., De Moor B., Verbeke K., Vanrenterghem Y., Evenepoel P. (2008). Free P-Cresol Is Associated with Cardiovascular Disease in Hemodialysis Patients. Kidney Int..

[31] Jing Y.J., Ni J.W., Ding F.H., Fang Y.H., Wang X.Q., Wang H.B., Chen X.N., Chen N., Zhan W.W., Lu L. (2016). P-Cresyl Sulfate Is Associated with Carotid Arteriosclerosis in Hemodialysis Patients and Promotes Atherogenesis in apoE−/− Mice. Kidney Int..

[32] Chang J.-F., Hsieh C.-Y., Liou J.-C., Liu S.-H., Hung C.-F., Lu K.-C., Lin C.-C., Wu C.-C., Ka S.-M., Wen L.-L. (2020). Scavenging Intracellular ROS Attenuates P-Cresyl Sulfate-Triggered Osteogenesis through MAPK Signaling Pathway and NF-κB Activation in Human Arterial Smooth Muscle Cells. Toxins.

[33] Gryp T., Huys G.R.B., Joossens M., Van Biesen W., Glorieux G., Vaneechoutte M. (2020). Isolation and Quantification of Uremic Toxin Precursor-Generating Gut Bacteria in Chronic Kidney Disease Patients. Int. J. Mol. Sci..

[34] Bochaton T., Paccalet A., Jeantet P., Crola Da Silva C., Cartier R., Prieur C., Jossan C., Bonnefoy-Cudraz E., Mewton N., Ovize M. (2020). Heat Shock Protein 70 as a Biomarker of Clinical Outcomes After STEMI. J. Am. Coll. Cardiol..

[35] Mechtouff L., Paccalet A., Crola Da Silva C., Buisson M., Mewton N., Amaz C., Bonnefoy-Cudraz E., Leboube S., Cho T.-H., Nighoghossian N. (2022). Prognosis Value of Serum Soluble ST2 Level in Acute Ischemic Stroke and STEMI Patients in the Era of Mechanical Reperfusion Therapy. J. Neurol..

[36] Verbeke F., Vanholder R., Van Biesen W., Glorieux G. (2022). Contribution of Hypoalbuminemia and Anemia to the Prognostic Value of Plasma P-Cresyl Sulfate and p-Cresyl Glucuronide for Cardiovascular Outcome in Chronic Kidney Disease. J. Pers. Med..

[37] Levey A.S., Stevens L.A., Schmid C.H., Zhang Y.L., Castro A.F., Feldman H.I., Kusek J.W., Eggers P., Van Lente F., Greene T. (2009). A New Equation to Estimate Glomerular Filtration Rate. Ann. Intern. Med..

[38] Kellum J.A., Lameire N., KDIGO AKI Guideline Work Group (2013). Diagnosis, Evaluation, and Management of Acute Kidney Injury: A KDIGO Summary (Part 1). Crit. Care.

[39] Fagugli R.M., De Smet R., Buoncristiani U., Lameire N., Vanholder R. (2002). Behavior of Non-Protein-Bound and Protein-Bound Uremic Solutes during Daily Hemodialysis. Am. J. Kidney Dis. Off. J. Natl. Kidney Found..

[40] Meert N., Schepers E., Glorieux G., Van Landschoot M., Goeman J.L., Waterloos M.-A., Dhondt A., Van der Eycken J., Vanholder R. (2012). Novel Method for Simultaneous Determination of P-Cresylsulphate and p-Cresylglucuronide: Clinical Data and Pathophysiological Implications. Nephrol. Dial. Transplant. Off. Publ. Eur. Dial. Transpl. Assoc.-Eur. Ren. Assoc..

