# Supplementary material for: The Uremic Toxin p-Cresyl Sulfate Is a New Predictor of Major Adverse Cardiovascular Events in Patients with ST-Elevation Myocardial Infarction
Source: Toxins (Basel). 2025 Dec 20;18(1):4. doi: 10.3390/toxins18010004 (PMC12846530; doi:10.3390/toxins18010004)
Supplement: Supplementary file 1 [file toxins-18-00004-s001.zip › toxins-3983265-supplementary.pdf]

Supplementary Table S1. Total plasma concentration of other uremic toxins in STEMI patients according to the occurrence of major cardiovascular events (MACE).

	Total Population	No MACE	MACE	p-value
N(%)	234 (100%)	209 (89%)	25 (11%)	
**Uremic toxins significantly associated with MACE**				
p-CS, µmol/L	2.74 (1.32-5.00)	2.70 (1.28-4.72)	4.13 (2.31-10.28)	**0.0036**
IS, µmol/L	2.27 (1.42-3.49)	2.19 (1.36-3.34)	3.14 (1.92-5.01)	**0.0154**
**Uremic toxins not significantly associated with MACE**				
IAA, µmol/L	0.759 (0.488-1.310)	0.768 (0.496-1.310)	0.652 (0.420-1.330)	0.602
CMPF µmol/L	3.32 (1.81 – 7.53)	4.32 (2.30 – 8.86)	3.32 (1.81 – 7.53)	0.187
p-CG, µmol/L	<LOD	<LOD	<LOD	-
Uric acid, µmol/L	402 (313 – 476)	403 (313 – 479)	373 (218 – 448)	0.501
Hippuric acid, mol/L	<LOD	<LOD	<LOD	-

Abbreviations: CMPF, 3-Carboxy-4-methyl-5-propyl-2-furanpropanoic acid, IAA, indole-3 acetic acid, IS, indoxyl sulfate, LOD, limit of detection, p-CG, p-cresyl glucuronide, p-CS, p-cresyl sulfate. Comparison of plasma level of association of uremic toxin level with MACE was estimated using Kaplan–Meier analysis and log-rank test.

Supplementary Table S2. Details of the 27 major adverse cardiovascular events (MACE) encountered by STEMI patients during the follow-up period according to their plasma pCS concentration.

MACE	Overall	Low pCS	High pCS
Death	5	0	5
Non-fatal reinfarction	7	4	3
Hospitalizations /Heart failure	12	6	6
Stroke	3	2	1
**Total**	**27**	**12**	**15**

Note that no significant difference was observed in the proportion of each class of event according to the level of p-CS (Fisher's exact test, p=0.1517). No difference was observed in the occurrence of each class of cardiovascular event according to the p-CS subgroup.

Supplementary Table S3. Correlation between uremic toxin levels and inflammatory biomarkers in STEMI patients.

	N	Spearman's r	p-value
**Indoxyl-sulfate**			
Neutrophiles/Leucocytes ratio	223	0.008 [-0.128 ; 0.143]	0.909
HSP-70	227	0.052 [-0.082 ; 0.185]	0.433
IL-6	229	0.140 [0.007 ; 0.269]	**0.034**
IL-8	229	0.175 [0.042 ; 0.301]	**0.008**
IL-10	229	0.189 [0.057 ; 0.315]	**0.004**
ST-2	228	0.126 [-0.008 ; 0.255]	0.058
MCP-1	229	0.247 [0.117 ; 0. 368]	**<0.001**
**p-Cresyl sulfate**			
Neutrophiles/Leucocytes ratio	223	0.073 [-0.062 ; 0.207]	0.275
HSP-70	227	0.071 [-0.063 ; 0.203]	0.284
IL-6	229	0.168 [0.036 ; 0.295]	**0.011**
IL-8	229	0.065 [-0.069 ; 0.197]	0.329
IL-10	229	0.219 [0.088 ; 0.342]	**<0.001**
ST-2	228	0.138 [0.005 ; 0.267]	**0.038**
MCP-1	229	0.076 [-0.058 ; 0.207]	0.254

Abbreviations : HSP-70, 70 kilodalton heat shock proteins, IL-6, interleukin-6, IL-8 interleukin-8, IL-10, interleukin-10, MCP-1, monocyte chemoattractant protein 1, ST2, Interleukin 1 receptor-like 1.

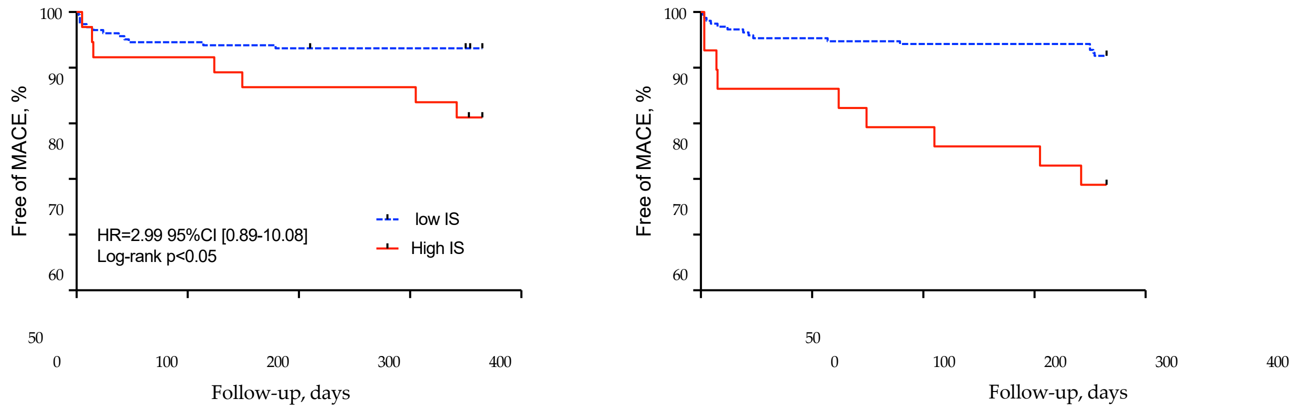

Number at risk

High IS	37	35	33	33	29
Low IS	184	175	173	172	168
	0	100	200	300	365

Number at risk

High pCS	29	26	24	23	20
Low pCS	191	183	181	181	176
	0	100	200	300	365

Supplementary Figure S1. High indoxyl sulfate (IS) and p-cresyl sulfate (p-CS) levels predict MACE in the sub-population of STEMI patients with eGFR 60 ml/min.1.73 m2 (i.e., no CKD at admission). Cut-offs determined using the Youden index of ROC curves. A. Kaplan–Meier curve for indoxyl sulfate (cut-off = 4.52 µmol/L). B. Kaplan–Meier curve for p-cresyl sulfate (cut off = 6.67 µmol/L).
